# Dimensionality reduction of independent influence factors in the objective evaluation of quality of experience

**DOI:** 10.1038/s41598-022-13803-z

**Published:** 2022-06-20

**Authors:** Fatima Skaka-Čekić, Jasmina Baraković Husić, Almasa Odžak, Mesud Hadžialić, Adnan Huremović, Kenan Šehić

**Affiliations:** 1 Faculty of Electrical Engineering, Department of Telecommunications, University of Sarajevo, Sarajevo, Bosnia and Herzegovina; 2 BH Telecom, Joint Stock Company, Sarajevo, Bosnia and Herzegovina; 3 Faculty of Science, Department of Mathematic, University of Sarajevo, Sarajevo, Bosnia and Herzegovina; 4 Department of Computer Science, Lund University, Lund, Sweden

**Keywords:** Electrical and electronic engineering, Scientific data

## Abstract

Big Data analytics and Artificial Intelligence (AI) technologies have become the focus of recent research due to the large amount of data. Dimensionality reduction techniques are recognized as an important step in these analyses. The multidimensional nature of Quality of Experience (QoE) is based on a set of Influence Factors (IFs) whose dimensionality is preferable to be higher due to better QoE prediction. As a consequence, dimensionality issues occur in QoE prediction models. This paper gives an overview of the used dimensionality reduction technique in QoE modeling and proposes modification and use of Active Subspaces Method (ASM) for dimensionality reduction. Proposed modified ASM (mASM) uses variance/standard deviation as a measure of function variability. A straightforward benefit of proposed modification is the possibility of its application in cases when discrete or categorical IFs are included. Application of modified ASM is not restricted to QoE modeling only. Obtained results show that QoE function is mostly flat for small variations of input IFs which is an additional motive to propose a modification of the standard version of ASM. This study proposes several metrics that can be used to compare different dimensionality reduction approaches. We prove that the percentage of function variability described by an appropriate linear combination(s) of input IFs is always greater or equal to the percentage that corresponds to the selection of input IF(s) when the reduction degree is the same. Thus, the proposed method and metrics are useful when optimizing the number of IFs for QoE prediction and a better understanding of IFs space in terms of QoE.

## Introduction

The future of mobile networks includes seamless combination and coexistent accesses of different standards, such as 4G, 5G, and 802.11 for delivering reliable, broadband superfast connectivity. A large amount of authentic network data is generated in these networks, which together with communication theory, data mining, statistical analysis, deep learning, and many other Artificial Intelligence (AI) technologies significantly improve the network performance, efficiency, and user experience with much-reduced network costs^1^. One of recognized challenges is utilization of Big Data analytics to provide user services with personalized Quality of Experience (QoE)^2^. The use of a large database comprising all the QoE Influence Factors (IFs), which can be used as input for the QoE model is limited in the literature^3^. Set of IFs, which has to be analyzed for appropriate estimation of QoE, varies depending on service type. Packet-switching technologies are particularly demanding for delivery of high-quality video^4^. As the dimension of the input set of IFs grows, it is crucial to recognize tools, which can appropriately deal with large datasets. AI and its sub-categories such as machine learning and deep learning have been evolving as a discipline, so this mechanism allows networks to be predictive and proactive^5^. Dimensionality reduction as part of machine learning is recognized as an essential step in the analysis of a large amount of data in a multidimensional context^6^. Different techniques exist for reducing data dimensions^7^.

Specification of methodology for identifying QoE IFs in an intuitive and systematic way includes categorization of IFs in different spaces^8^ and initial multidimensional space can be divided into several subspaces. The multidimensional context used in this study is related to the number of IFs used as input for QoE modeling. The number of input IFs uniquely corresponds to the number of dimensions. Adding a new IF to the model increases the problem dimension, but also the accuracy of the model. After the initial set of input IFs is defined, different dimensionality reduction techniques can be applied depending on the type of input IFs. Dimensionality reduction techniques can be categorized into linear and non-linear; supervised and unsupervised; feature selection and feature extraction. The differences between these techniques lie in their different motivations and objective functions^9^, as well as in the type of input data. Feature extraction transforms data into features suitable for modeling whereas feature selection removes unnecessary features^10^. Dimensionality reduction is useful for data modeling, compression and visualization. An initial step for a comprehensive analysis of dimensionality reduction in QoE modeling includes a meta-analytical overview of applied dimensionality reduction methods in QoE modeling.

Active subspaces method (ASM)^11–15^ is a gradient-based dimensionality reduction technique for feature extraction of an independent set of input parameters. In QoE modeling, the calculation of gradients for most IFs is inadequate since they are a discrete or categorical variable. Calculation of gradients can also be a challenge for some IFs since the explicit functional dependence of QoE in terms of IFs is mostly unknown. This is the motivation for the introduction of ASM method modification to overcome these problems and thus creating the possibility to apply these kinds of dimensionality reduction methods to problems with a wider range of input IFs compared to the original ASM. The application of the modified ASM method is not limited to QoE management but can be applied in other areas where the same issues are identified. The introduction of categorical variables for many models can be crucial since a categorical variable such as gender^16,17^ in real applications should not be neglected. Outputs of ASM and mASM are comparable with global sensitivity analysis metrics.

QoE modeling is most often based on a set of IFs that have been empirically shown to have the greatest impact on QoE. Ignoring IFs that do not intuitively affect QoE much belongs to dimensionality reduction where no previous analysis of available IFs has been performed, but they have been ignored based on experience. In order to compare the methods of dimensionality reduction and previous practice of selection specific IFs, the paper introduces a new metric that aims to compare the two approaches. Besides the mathematical approach, comparative analysis of all methods and metrics are done for a selected set of influence factors with an objective evaluation of QoE. Objective evaluation of QoE differs from subjective evaluation which can be time consuming and expensive with no real time possibility to monitor video quality as it is mostly conducted by asking users to score the quality of video^18^.

According to the abovementioned, the objective of this study can be summarized as:

1. Meta-analytical overview of used dimensionality reduction methods applied to the input set of IFs in QoE modeling;
2. Introduction of modification of ASM method with variance/standard deviation (STD) as a measure of function variability;
3. Introduction of new metrics for quantitative measure of the amount of function variability of the linear combination of IFs to compare feature selection and feature extraction approach;
4. Numerical analysis with performance analysis of ASM and mASM.

To accomplish the given objectives, we organize the rest of the paper as follows. Section “Releated work” presents the related work considering dimensionality reduction in QoE modeling. Section “Problem statement” gives a problem statement, section “Overview of existing method and metrics” the theoretical background, and section “Introduction of new method and metrics” the mathematical model of the method and metrics introduced in this paper. Section “Numerical analysis” presents the numerical results obtained for ASM and mASM method. Section “Discussion” discusses obtained results and outlines recommendations for future work. Section “Conclusion” concludes the paper.

## Releated work

QoE can be defined as: “the degree of delight or annoyance of the user of an application or service. It results from the fulfilment of his or her expectations with respect to the utility and/or enjoyment of the application or service in the light of the user’s personality and current state^19^.” Factors which influence QoE represent “any characteristic of a user, system, service, application, or context whose actual state or setting may have influence on the Quality of Experience for the user^19^.” Set of IFs can be described in the form of a QoE feature by the end user. IFs are grouped into categories: Human IF (HIF), System IF (SIF), and Context IF (CIF). Correlation modeling between multiple IFs and precise QoE prediction can be a demanding task because the exact model requires an analysis of a large number of IFs. The multidimensional nature of QoE represents a challenge for modeling, since the QoE prediction techniques have limitations in terms of input complexity and possible interdependence of IFs. Large input complexity leads to unacceptable computational complexity and time-consuming problems. According to^20^, some researchers find sufficient to correlate the QoE to a single network Quality of Service (QoS) parameter, e.g. delay or throughput, whereas others argue that multiple QoS parameters affect the QoE and need to be considered in tandem as features in a QoE/QoS correlation model. Consideration of only one or two QoE IFs is generally not sufficient for accurate QoE assessment^21^. On the contrary, QoE should be considered in all its dimensions considering as many IFs as possible (and relevant)^21^.

According to the above mentioned, it can be concluded that the number of IFs, which will be used for QoE evaluation is preferable to be higher. Combination of all IFs gives the best performance over most regression models^22^. This suggests that a successful QoE prediction model should consider diverse QoE-aware IFs in order to better predict QoE^22^. After collecting IFs, they can be used as input for the QoE evaluation algorithm, which can lead to unacceptable complexity. Real world data analysed by data mining algorithms can include a large number of irrelevant or redundant features for a learning algorithm to handle them efficiently^23^. For this reason, dimensionality reduction is used in order to deal with big dataset collected from the mobile network traffic^3^. Extracting features and deriving/analysing user's experiences from a large amount of data should be a focus of future research^24^. Statistical methods such as Principal Component Analysis (PCA) may be promising to identify the perceptual dimensions^25^. Furthermore, dimensionality reduction enables the selection of subsets of features that are useful to build a good predictor, especially when some of the IFs are redundant^3^. In most cases, preparation of data and descriptive analytics work together to enable better understanding of the big dataset and prepare them for the modeling stage ^3^. But there is a lack of generalized and powerful mining tools for datasets with multiple dimensions as videos and images^26^. Dimensionality reduction is an important step for data preprocessing in data mining and knowledge discovery^27^. Dimensionality reduction techniques can be used for eliminating interdependence of IFs for methods, which require independent inputs for estimating QoE^27,28^.

Successful QoE management requires deep understanding of QoE IFs and quality of perception^21^. The QoE management process may be divided into three general steps: (1) QoE modeling, (2) QoE monitoring and measurements, and (3) QoE optimization and control. Aims of different QoE management process steps can be summarized as follow: (1) QoE modeling specifies the relationship between different measurable QoE IFs and QoE dimensions, (2) QoE monitoring and measurement are used for acquisition of data related to the network environment and conditions, terminal capabilities, user, context and application/service specific information and its quantification, (3) QoE optimization and control have intention to optimize service delivery with (potentially) continuous and dynamic delivery control in order to maximize the end-user’s satisfaction and optimally utilize limited system resources^21^. According to the specified aim, this paper is focused on the QoE modeling step. The QoE modeling step may be further divided into three general steps: (1) Data collection, (2) Data preparation, and (3) Data modeling. Data collection can generally be implemented based on experimental and simulation approaches where collected data can be mutually correlated or independent. The data preparation step is introduced in QoE modeling to deal with unacceptable complexity^3^. The data modeling step consists of predictive and prescriptive analytics^3^. Predictive analytics involves the process of modeling perceived QoE or Mean Opinion Score (MOS). The prescriptive analytics takes advantage of the results obtained from both the descriptive and predictive analytics to decide the best decision or action^3^. Descriptive, predictive, and prescriptive methods of big data analytics are given in^2,29^. This paper is focused on the Data preparation step. The data preparation step may be further divided into the following steps: (1) Data preprocessing, (2) Data exploratory analysis, and (3) Dimensionality reduction^3^. Data preprocessing contains cleaning, integration, and data transformation. Data exploratory analysis uses statistical techniques for understanding the dataset. Dimensionality reduction enables the selection or extraction of features to transform raw data into representation data^30^. This paper is focused on the Dimensionality reduction step. The dimensionality reduction step includes (1) Feature selection and (2) Feature extraction. Feature selection aims to select the most relevant features, whereas extraction combines the features into a reduced set of features^3^. Different terminology is used. According to^12^, dimensionality reduction methods are divided into two groups, methods based on subsets and methods based on subspaces (i.e. selection and extraction). Manifold learning and embedded learning are synonyms for feature extraction. Feature extraction (subspace learning) includes (1) Unsupervised feature extraction, and (2) Supervised feature extraction. Unsupervised feature extraction includes PCA, Singular Value Decomposition (SVD), Latent Semantic Analysis (LSI), Locality Preserving Projections (LPP), Independent Component Analysis (ICA), Projection Pursuit (PP), Kernel PCA, Multidimensional Scaling (MDS), Isomap, Locally Linear Embedding (LLE), Self-Organizing Map (SOM), t-distributed Stochastic Neighbor Embedding (t-SNE). Supervised feature extraction includes Linear Discriminant Analysis (LDA), Learning Vector Quantization (LVQ)^31^. The main objective of feature extraction is to get subspaces where more inference and efficient learning can be obtained^30^. Feature selection includes (1) Filter methods, (2) Wrapper methods, and (3) Embedded methods^32^. Filter methods rank features using criteria. Wrapper methods take advantage of a learning algorithm as a part of the feature selection. Embedded methods combine the qualities of filter and wrapper methods.

According to listed dimensionality reduction methods, first an overview of which methods are suitable for an independent set of IFs is given, and then an overview of the application of all the above methods to the input set of IFs in QoE modeling is given.

### Dimensionality reduction methods appropriate for an independent set of IFs

Since listed feature extraction methods are based on assumptions that features are not uncorrelated and that they share some information^33^, it is necessary to examine whether these methods are appropriate for application to an independent set of input IFs in QoE modeling. Unsupervised feature extraction methods seek for correlation between input IFs to perform the reduction. PCA is a method where linear transformations of correlated variables are generated to produce relatively uncorrelated variables^11^. Other methods are highly interrelated and in special cases equivalent to PCA^34^ so it explains the initial statement that these methods are introduced to remove redundancy in input data^33^. LSA is designed for text documents with the aim to learn text semantic representation. PP predefines the objective function called projection pursuit index and projection is done by maximizing this function. ICA performs linear transformation of input correlated data in such a way that outputs are independent. LPP relies on the linear approximation of the Laplacian Eigenmaps with an aim to preserve distances between samples when projecting data to lower space. Laplacian Eigenmap uses similarity of neighbour samples. KPCA is non-linear PCA so first mapping the data is done using a nonlinear function^35^. MDS uses distance metric so it becomes PCA when Euclid distance is used. Isomap and LLE outweigh the disadvantage of PCA of not capturing the possible non-linear essence of pattern. According to this, Isomap, LLE, and Laplacian Eigenmaps can be considered as special cases of KPCA, whereas KPCA is identical to PCA when linear kernel is used.

Since dimensionality reduction of an independent set of IFs requires information of QoE change, supervised methods are appropriate for this task. Supervised methods LDA and LVQ are applicable to an independent set of inputs and can be used for weight determination. Besides dimensionality reduction, LDA is a classification approach, and it relies on the mean of samples and covariance matrices computed from training sample from different groups^36^. LDA determines weighted coefficients for inputs in a way to give the best separations between known groups of observations. According to this, application LDA and ASM on the input set of IFs gives different weighted coefficients with different application possibilities. LDA uses classes of QoE and transformation of original space of input IFs is made in a way to get a projection maximizing the ratio between different QoE classes while minimizing the ratio within QoE classes. So, besides dimensionality reduction (data preparation), LDA is used for data modeling. ASM, in contrast, sees QoE as a function of input IFs where QoE values need not be a constraint to a limited set of values and transformation of original space of input IFs is made in a way that the first dimension contains the highest change of QoE, each following dimension is less important, and that for the last dimensions there are no major changes of the QoE function or the changes are zero. So, the basic disadvantage of LDA in comparison to ASM is that LDA is limited to classification problems only. LDA does not address the problem of continuous target variables, so these techniques are not applicable to the family of regression problems^37^. Similar to LDA, LVQ also performs classification using distances between input vectors and its advantage is classification accuracy^31^, but it retains the same disadvantage as LDA in comparison to ASM. According to the above, application of ASM in the Data preparation phase enables selection of reduction degree according to desired accuracy and acceptable complexity, a better understanding of input IFs with no restriction later to Data modeling classification methods only.

### Applied dimensionality reduction methods on the input set of IFs

Neglecting approaches where IFs are ignored based on experience, according to above-mentioned methods for feature extraction and feature selection, Table 1 depicts related studies that have used dimensionality reduction methods for data preparation in the process of modeling perceived QoE. From references review (Table 1), the following can be noticed:

- Dimension of IF vector ranges from 3 to 5200, so it can be concluded that the application of the dimensionality reduction technique is not limited by the dimension of the input vector (which can be subject to further consideration depending on the type of dimensionality reduction technique).
- Used IFs can be categorized into SIF (90.26%), then CIF (8.11%), and HIF (1.63%), so it can be noticed that SIFs are dominantly used to form the vector of IFs.
- The following feature extraction techniques are present: PCA (45%), Factor Analysis (FA) (23%), LDA (14%), MDS (9%), Stochastic Neighbor Embedding (SNE) (4.5%), and LLE (4.5%).
- The following feature selection techniques are present: Filter methods (40%), Wrapper methods (30%), and Embedded methods (30%).
- Feature extraction techniques are used for the creation of a new reduced dimension, which is the combination of all input IFs^27,28,38–48^, the selection of the most important IFs^6,44,49^, elimination of the interdependence of input vector^27,28,50^, QoE prediction based on classification^51–53^. There are some special application aims such as determining the weighted coefficients to be used within the mathematical model^50^. The combined application of the feature selection and feature extraction method is given in^53^ where LDA is used for QoE prediction.
- The achieved degree of dimensional reduction after the Data preparation phase ranges from 99.6% (input: 5200, output: 20)^47^ to the example where the dimensionality reduction technique was not applied to achieve the reduction (0% reduction)^50^. The reduction degree that the dimensionality reduction technique can accomplish, is essentially conditioned by the selection of the input IFs, where the selection of IFs can be such that more IFs describe the same change in the system, so they are highly interdependent.

**Table 1:** Review of related work considering the applied dimensionality reduction methods in QoE modeling.

Ref	Dim. reduction method	Dimension of input IFs				Dim. of reduced IFs	Reduction degree
		SIF	CIF	HIF	Sum		
**Feature extraction**							
^38^	PCA	239	13	2	254	5	5/254
	MDS						
	SNE						
^28^	wPCA	9			9	3	3/9
^6^	PCA	9	2		11	5	5/11
^39^	FA	14			FR:14	3	3/14
		10			NRcl:10	3	3/10
		7			NRsp:7	3	3/7
^40^	FA	14			FR:14	3	3/14
		10			NRcl:10	3	3/10
		7			NRsp:7	3	3/7
^41^	FA	14			FR:14	4	4/14
		10			NRcl:10	4	4/10
		7			NRsp:7	4	4/7
^50^	PCA	1		4	5	5	5/5
^42^	FA VRM	9	3		12	3	3/12
^43^	MFA	1			6 values of one IF	2	2/6
^44^	PCA	3			3	2	2/3
		3			3	3	3/3
^45^	PCA	5			5	2	2/5
^46^	PCA	5	17		22	5	5/22
^49^	PCA	4	1		5	2	2/5
^27^	PCA	10			10	3	3/10
^47^	PCA		1		5200 values of one IF	20	20/5200
						40	40/5200
	LLE					60	60/5200
						80	80/5200
^48^	MDS	5			5	4	4/5
^51^	LDA	40			40	–	–
^52^	LDA	3			840 samp. of 3 IF	–	–
					552 samp.of 3 IF		
^53^	LDA	6	3	2	UFS: 5/6/11	–	–
					RFE: 2/10/11		
					DT: 5		
**Feature selection**							
^54^	Embedded:M5P	19			19	6	6/19
^55^	Embedded: C4.5	9	1		10	YTB MOS: 5	5/10
						FCB MOS: 1	1/10
						Gmaps MOS: 3	3/10
						YTB ACC: 6	6/10
						FCB ACC: 4	4/10
						Gmaps ACC: 4	4/10
^56^	Wrapper: SVM	Raw: 9			9	3–5	3–5/9
		Extended: 27			27	–	–
^57^	Wrapper: RC	11			11	–	–
^53^	Filter: UFS	6	3	2	11	6	6/11
	Wrapper: RFE					2	2/11
	Embedded: DT					2	2/11
^58^	Filter: SCA	–	–	–	89	11	11/89
^59^	Filter: IG	–	–	–	208	97	97/208
	Filter: CFS					18	18/208

*CFS* Correlation based feature selection, *DT* Decision Trees, *FA* Factor Analysis, *FCB* Facebook, *FR* Full Reference, *IG* Information Gain, *LDA* Linear Discriminant Analysis, *LLE* Local Linear Embedding, *MDS* Multidimensional Scaling, *MFA* Multiple Factor Analysis, *NRcl* Non Reference client, *NRsp* Non Reference Service Provider, *PCA* Principal Component Analysis, *RC* Regression coefficients, *RFE* Recursive feature elimination, *SCA* Spearman correlation analysis, *SNE* Stochastic Neighbor Embedding, *SVM* Support Vector Machine, *UFS *Univariate feature selection, *YTB* YouTube, *wPCA* weighted Principal Component Analysis, *VRM* Varimax Rotation Method.

## Problem statement

For the formal introduction of methods and metrics, general notation is as follows. Function $f=f(x)$ is defined on a $N$-dimensional hypercube, i.e. $x=\left[{x}_{1},{x}_{2},\dots ,{x}_{N}\right]\in {\left[-\mathrm{1,1}\right]}^{N}$ with uniform probability density function $\rho \left(x\right)={2}^{-N}$ for $x\in {[-\mathrm{1,1}]}^{N}$ and zero elsewhere. The mean and variance of function $f$ are given by ${\mathbb{E}}\left[f\right]=\int f\left(x\right)\rho \left(x\right)dx$ and $Var[f]=\int {\left(f\left(x\right)-{\mathbb{E}}\left[f\right]\right)}^{2}\rho \left(x\right)dx$. Gradient of $f$ is denoted by $\nabla f(x)$ with partial derivatives denoted by $\frac{\partial f}{\partial {x}_{i}}(x)$, for $i=\mathrm{1,2},\dots ,N$. Let define ${f}_{i}\left({x}_{i}\right)=f({x}_{1},{x}_{2},\dots {x}_{i},\dots ,{x}_{N})$ where ${x}_{i}$ is sampled through *i*-th direction while others ${x}_{o}, o\in \left\{\mathrm{1,2},\dots ,N\right\}\backslash \left\{i\right\}$ are fixed. The subscript $\left({x}_{i}\right)$ represents $i$-th input whereas the superscript $\left({x}^{s}\right)$ represents *s*-th sample, therefore $\left({x}_{i}^{s}\right)$ is $s$-th sample of $i$-th input.

Dimensionality reduction in QoE modeling implies function $QoE$ defined on a $N$-dimensional set of IFs ${\left\{{IF}_{i}\right\}}_{i=1}^{N}$ where ${IF}_{i}$ is the $i-th$ IF. In special case $IF\in {\mathbb{R}}^{N}$ whereas same IFs can be categorical or discrete variables. Dimensionality reduction technique creates a new set of influence factors ${\left\{{{IF}_{new}}_{j}\right\}}_{j=1}^{M}$, and can be expressed as mapping $IF\to {IF}_{new}$ where $IF$ form $N$-dimensional space of original inputs, and ${IF}_{new}$ form $M$-dimensional space where $M\le N$. $QoE$ is quantity of interest and can be presented as a function of the initial set of input IFs: $QoE=f\left(IF\right)$. The goal of dimensionality reduction in QoE modeling can be expressed as a better representation of available data with new dimension $M$. In feature selection, $M\le N$ and ${\left\{{{IF}_{new}}_{j}\right\}}_{j=1}^{M}\subseteq {\left\{{IF}_{i}\right\}}_{i=1}^{N}$, so selected set of ${IF}_{new}$ is an inclusive subset of the input IFs. In feature extraction, ${\left\{{{IF}_{new}}_{j}\right\}}_{j=1}^{M}$ is just in special case a subset of ${\left\{{IF}_{i}\right\}}_{i=1}^{N}$, but usually ${IF}_{new}=g(IF)$. The special case of this mapping is linear transformation so ${{IF}_{new}}_{j}=\sum_{i=1}^{N}{w}_{ij}{IF}_{\mathrm{i}}$. Weighted coefficients for ${{IF}_{new}}_{j}$ form $N$-dimensional row vector ${\left\{{w}_{i}\right\}}_{i=1}^{N}$, so $W : = {\left[{w}_{1}, \dots ,{w}_{M}\right]}^{T}\in {\mathbb{R}}^{MxN}$. $W$ is called weighted coefficients matrix.

Special cases of a linear transformation are feature selection and even weight distribution. For feature selection, weighted coefficients for the selected set of IFs ${\left\{{IF}_{i}\right\}}_{i=1}^{M}$ are 1, ${w}_{ii}=1$, while others weighted coefficients are 0, ${w}_{ij}=0,i\ne j$. Even weight distribution is a model where all IFs are nearly equally important with weighted coefficients ${w}_{ij}\approx 1/N$ for all $i=1,\dots ,N, j=1,\dots ,N$.

$QoE$ as a function defined on $N$-dimensional space can be flat or it has negligible changes in some directions. These directions are optimal for dimensionality reduction since by neglecting these directions the slightest loss of information about the changes of $QoE$ will be realized. The objective of linear transformation feature extraction is the determination of weighted coefficients in a way that the first $M {IF}_{new}$ vectors contain the highest changes of $QoE$ whereas for other $N-M {IF}_{new}$ vectors $QoE$ is flat or has negligible changes of $QoE$. Dimension of reduced space $M$ can be chosen from 1 to $N$, $1\le M\le N$, where for $M=N$ rotation of initial space is done without reduction. Choosing $M<N$, initial $N$ dimensional input space is reduced to $M$ dimensional space.

**Figure 1:**
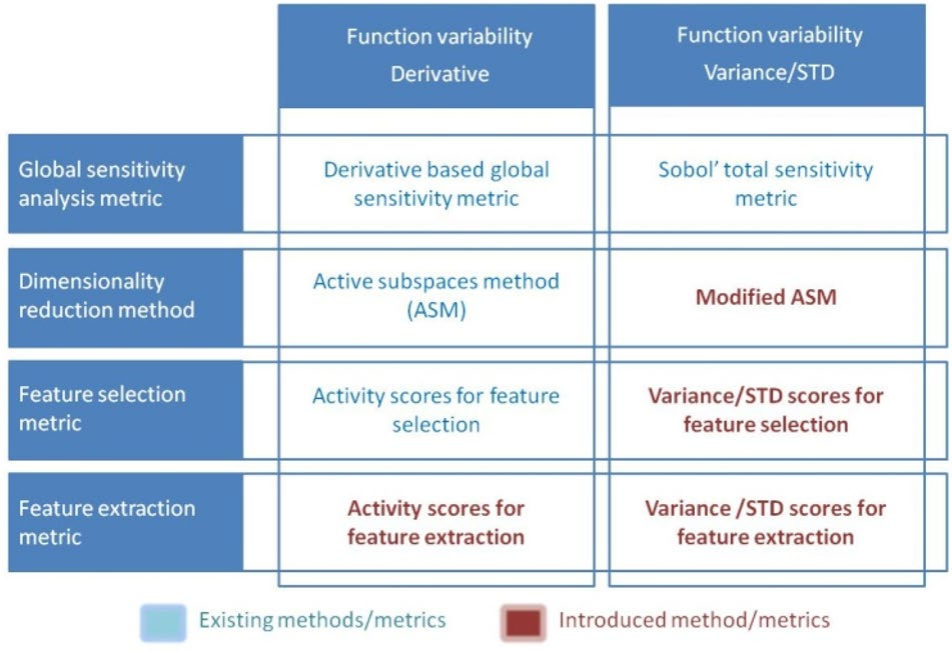
Overview of derivative and variance/STD based methods and metrics.

**Function variability** can be measured through the first derivative as slope or “rate of change” of a function, and through the variance/STD as a measure of how a function is spread out. Finding derivative of the function in points gives small scale behaviour of the function near these points. Variance/STD measures the variability of function from the average or mean of the function. The appropriate choice of measure of variability depends on the application^60^. Directions with the first order derivatives of function equal to zero or with variance/STD equal to zero can be used for dimensionality reduction since they determine regions where the function is flat. Figure 1 gives an overview of methods and metrics based on which measure of function variability is used. Red marked method and metrics are introduced in this paper.

**Global sensitivity analysis metric** uses derivatives and variance as a measure of function variability where Derivative based global sensitivity metric uses derivatives and Sobol’s total sensitivity metric uses variance as a measure of function variability. Sensitivity analysis is a study connected to feature selection since it determines how the variations in the output of a model can be apportioned to different sources of variation^61^. Sensitivity metrics are classified as local—nominal parameter value is small changed for measurement of model’s response and global—measurements of importance of each variable over a range of parameters. The basic approach is varying the input parameters to a model to see how the output is affected^11^. Commonly used sensitivity metrics are above mentioned Derivative based global sensitivity metric and Sobol’s total sensitivity metric^60^.

**Dimensionality reduction method** ASM uses derivatives as a measure of function variability. Outputs of ASM are eigenvalues and eigenvectors used to form ${IF}_{new}$. Eigenvectors contain information of influence of particular IF on $QoE$ per dimension, so using the ASM approach, weighted coefficients determination can be done where every reduction degree $(M\le N)$ is determined with the corresponding eigenvalue ${\lambda }_{M}$. The mean squared directional derivative of $QoE$ with respect to the eigenvector ${w}_{i}$ is equal to the corresponding eigenvalue ${\lambda }_{i}$^12^ so for smallest ${\lambda }_{i}\approx 0$, changes of $QoE$ are zero or negligible. Weighted coefficients matrix $W$ can be interpreted as follow: first column ${\left\{{w}_{i1}\right\}}_{i=1}^{N}$ contains first eigenvector components which represent weighted coefficients of particular IFs containing information about its influence, so reducing initial $N$-dimensional space to $1$-dimensional space, influence of particular IF is determined by these weighted coefficients. Particular row $i$ of weighted coefficients matrix ${\left\{{w}_{ij}\right\}}_{j=1}^{M}$ corresponds to particular IF and contains information of the portion of influence of particular IF per dimensions.

First row is connected to the first IF, so first element ${w}_{11}$ is influence of ${IF}_{1}$ on $QoE$ in first dimension, ${w}_{12}$ influence of ${IF}_{1}$ on $QoE$ in the second dimension, while ${w}_{1N}$ is influence of ${IF}_{1}$ on $QoE$ in least important dimension where $QoE$ is nearly flat.

Determining gradients when using ASM can be a challenge since the explicit functional dependence of $QoE$ in terms of IFs is mostly not known. Usually, approximation needs to be used, commonly finite differences method. Also, gradients may not be used when variables are categorical or discrete. So, methods that overcome these problems are of importance. We introduce mASM as a dimensionality reduction method which uses variance/STD as a measure of variability which completely overcomes the problem of finding gradients and it is applicable to a wider range of input IFs. The use of variance in QoE modeling to describe the relationship of independent inputs and QoE exists through statistical analysis of ANalysis Of VAriance (ANOVA)^62–66^ whereby in this paper variance/STD is used for dimensionality reduction as input to SVD analogously as gradients are used as input to SVD in the case of ASM. ANOVA is also used in dimensionality reduction, but as a criterion for feature selection^67^. mASM differs from PCA and its supervised modification^68^ where variance is calculated over inputs while in the mASM variance/STD is calculated over the function of inputs. Modification of ASM is also given in^69^ where modification includes usage of average of gradients which does not overcome the issue of applicability on categorical variables and it requires calculation or approximation of gradients which may be difficult or inadequate for some IFs.

**Feature selection metric** Activity scores for feature selection (ASFS) is a sensitivity metric obtained from the ASM procedure which quantifies how much each IF describes change of $QoE$. ASFS is comparable with the Derivative based global sensitivity metric since both are based on derivatives and according to^60^ ASFS is equal to the Derivative based global sensitivity metric when $M=N$. Analogously to ASFS, in this paper, we introduce Variance/STD scores for feature selection (VSFS) as an output from mASM and compare it with global Sobol’s total sensitivity metric.

**Feature extraction metric** Activity scores for feature extraction (ASFE) is introduced in this paper which quantifies how much weighted combination of IFs describes changes of $QoE$. Using ASFE and ASFS it is possible to compare feature extraction and feature selection approach for every reduction degree. Analogously to ASFE, in this paper, we introduce Variance/STD scores for feature extraction (VSFE) as an output from mASM with the possibility to compare it with VSFS.

**Figure 2:**
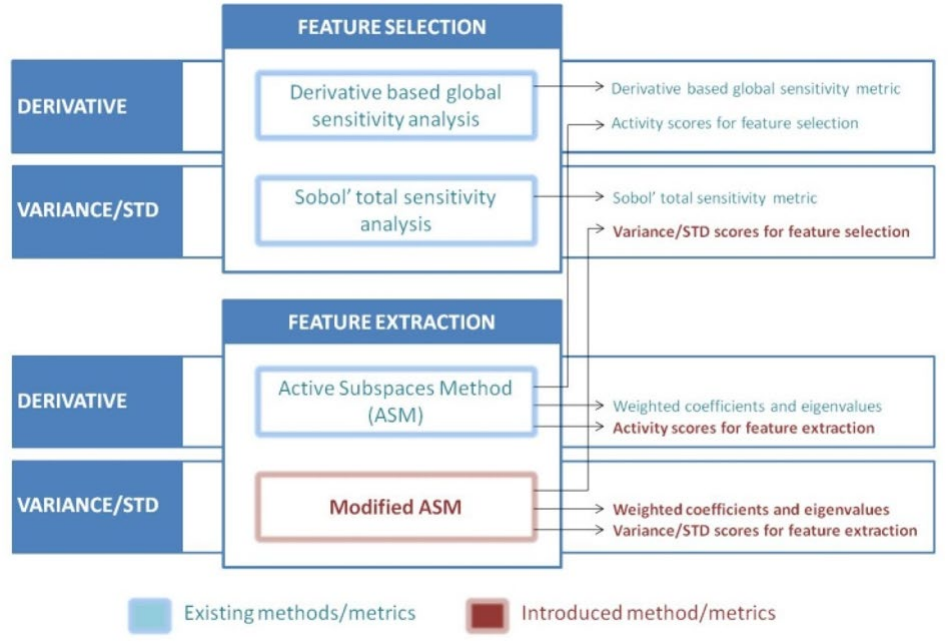
Overview of derivative and variance/STD based methods and metrics for feature selection and feature extraction.

Overview of above-mentioned methods and metrics is given in Fig. 2 divided by type of dimensionality reduction feature selection and feature extraction where outputs of methods are indicated. For further explanation of the red marked method and metrics, the following chapter gives a brief overview of the existing methods and metrics from Figs. 1 and 2.

## Overview of existing method and metrics

According to Fig. 1, in this chapter overview of existing methods and metrics is given. Since determining gradients is not a problem only for QoE modeling, and also discrete and categorical inputs are not present in IFs space only, therefore we will use general notation in the sequel.

### Global sensitivity analysis metric: Sobol’ total sensitivity metric

Sobol’ total sensitivity analysis is derived from the functional ANOVA decomposition or the variance-based decomposition. Let $i\in \left\{1,\dots ,N\right\}$ and ${S}_{i}$ is a set of subsets containing the index $i$. ANOVA decomposition of function is $f\left(x\right)=\sum_{u\subseteq \{1,\dots ,N\}}{f}_{u}(x)$. Sensitivity metric is total effect index^60,70^:

1 $${\tau }_{i}=\frac{\sum_{u\subseteq {S}_{i}}Var[{f}_{u}]}{Var[f]}.$$

Jansen’s formula for approximation of ${\tau }_{i}$ is:

2 $${\tau }_{i}\approx \widehat{{\tau }_{i}}=\frac{1}{2{N}_{p}{{\widehat{\sigma }}_{f}}^{2}}\sum_{p=1}^{{N}_{p}}{\left({f(A)}_{p}-{f({{A}_{B}}^{(i)})}_{p}\right)}^{2},$$

where ${{\widehat{\sigma }}_{f}}^{2}$ is variance of points of the ${N}_{p}$ evaluation of function $f$ in $f(A)$ which approximates the variance of $f$. Matrices $A$ and $B$ contains $2{N}_{p}$ samples of ${x}_{i}$. Matrix ${{A}_{B}}^{(i)}$ is a matrix $A$ whose *i-*th column is replaced by *i-*th column of $B$.

### Global sensitivity analysis metric: derivative based global sensitivity metric

Derivative based global sensitivity analysis is based on output changes to small variety in models inputs through derivative analysis. This metric can be expressed as^60,71^

3 $${v}_{i}=\int {\left(\frac{\partial f}{\partial {x}_{i}}\left(x\right)\right)}^{2}\rho \left(x\right)dx,i=1,\dots ,N.$$

Monte Carlo estimation of ${v}_{i}$ for ${N}_{p}$ points is

4 $${v}_{i}\approx \widehat{{v}_{i}}=\frac{1}{{N}_{p}}\sum_{j=1}^{{N}_{p}}{\left(\frac{\partial f}{\partial {x}_{i}}\left({x}_{j}\right)\right)}^{2}.$$

### Dimensionality reduction method: active subspaces method

In general, ASM is a feature extraction method where each direction is determined by a set of weighted coefficients that defines a linear combination of all inputs. Reduction is based on an estimation whether the function prediction changes as the inputs move along a particular direction. Direction can be ignored in the parameter study if there is no change along it or the change is negligible. The assumptions for the application of ASM are as follows: simulation model is with $N$ defined inputs and measured scalar quantity of interest, ranges are specified for each of the independent input parameters, and available resources for running the simulation multiple times. The required number of simulations for this algorithm is

5 $${N}_{p}={\alpha }_{of}M\mathrm{log}N,$$

where the real constant ${\alpha }_{of}$ is an oversampling factor that is usually chosen in the range between 2 and 10^12^, $M\le N$ is the number of eigenvalues to be used in the model after reduction. Eigenvalues are used to determine the size of the active subspace, based on gaps between eigenvalues, whereas the corresponding eigenvectors define the active subspace. The theory behind active subspaces begins with a matrix $C$ which is defined as

6 $$C=\int ({\nabla }_{x}f){({\nabla }_{x}f)}^{T}\rho dx,$$

where $f$ is the quantity of interest in a given computational model, the gradient of $f$ is taken in accordance with the model parameters, and ρ is the probability density function. Input column vector is.

7 $${\nabla }_{\mathrm{x}}\mathrm{f}\left(\mathrm{x}\right)=\left[\begin{array}{c}\frac{\partial \mathrm{f}}{\partial {\mathrm{x}}_{1}}(\mathrm{x})\\ \vdots \\ \frac{\partial \mathrm{f}}{\partial {\mathrm{x}}_{\mathrm{N}}}(\mathrm{x})\end{array}\right]$$

Since $C$ is a symmetric matrix, eigendecomposition is possible and given by

8 $$C=W\Lambda {W}^{T},$$

where $$\Lambda$$ is

9 $${\Lambda } = diag\left( {\lambda_{1} , \ldots ,\lambda_{N} } \right),\;\;\lambda_{1} \ge \ldots \ge \lambda_{N} \ge 0,$$

and $W$ is an orthogonal matrix whose columns are orthonormal eigenvectors ${w}_{i}, i=1,\dots ,N$ that correspond to ${\lambda }_{1},\dots ,{\lambda }_{N},$ respectively. When eigendecomposition is performed, it is possible to separate eigenvalues and eigenvectors in the following way

10 $$\Lambda =\left[\begin{array}{cc}{\Lambda }_{1}& \\ & {\Lambda }_{2}\end{array}\right], W=\left[\begin{array}{ll}{W}_{1}& {W}_{2}\end{array}\right],$$

where ${\Lambda }_{1}$ contains “large” eigenvalues, and ${\Lambda }_{2}$ “small” eigenvalues, ${W}_{1}$ contains eigenvectors assigned to “large” eigenvalues, and ${W}_{2}$ contains eigenvectors assigned to “small” eigenvalues. Active subspace is obtained from gradients $\nabla f(x)$ and determination of the active subspace requires the ability to calculate gradients or gradient approximations at any point $x$ from the domain under consideration. In the case where the gradients are unknown, and the simulation is manageable, it is possible to use the approximate values of gradients through the method of finite differences. In this case, the required number of simulations is

11 $${N}_{sim}={\alpha }_{of}M(N+1)\mathrm{log}N.$$

Approximation of eigenvalues and eigenvectors of the matrix $C$ defined by (6) can be done using a random sampling algorithm.

**Algorithm 1: ASM**

1. Take ${N}_{p}={\alpha }_{of}M\mathrm{log}N$ independent points $\left\{{x}^{1},{x}^{2},\dots ,{x}^{{N}_{p}}\right\}$ from the domain of interest according to the probability density function $\rho (x)$.
2. For each point ${x}^{p}$, calculate gradients ${\nabla }_{x}{f}^{p}=\nabla f\left({x}^{p}\right)$.
3. Form input column vector (7) and approximate
  12 $$\widehat{C}=\frac{1}{{N}_{p}}\sum_{i=1}^{{N}_{p}}{\nabla }_{x}{f}^{p}{\nabla }_{x}{{f}^{p}}^{T}.$$
4. Compute the eigendecomposition
  13 $$\widehat{C}=\widehat{W}\widehat{\Lambda }{\widehat{W}}^{T}$$
  where $\widehat{\Lambda }=diag({\widehat{\lambda }}_{1},\dots ,{\widehat{\lambda }}_{N})$ is the diagonal matrix of eigenvalues arranged in a decreasing order and $\widehat{W}$ is the matrix of eigenvectors ordered accordingly. The last step of computation of eigendecomposition is equal to SVD of the matrix^12^.

14 $$\frac{1}{\sqrt{{N}_{p}}}\left[{\nabla }_{x}{f}^{1}\dots {\nabla }_{x}{f}^{N}\right]=\widehat{W}\sqrt{\widehat{\Lambda }}\widehat{V},$$

where singular values are the square roots of the eigenvalues, and $\widehat{W}$ are the eigenvectors.

### Feature selection metric: activity scores for feature selection

ASFS is a sensitivity metric from the eigenpairs according to^60^ and it is used to rank the importance of inputs, so ASFS can be expressed as:

15 $${{\alpha }_{FS}}_{i}={\alpha }_{{FS}_{i}}\left(M\right)=\sum_{j=1}^{M}{\lambda }_{j}{{w}_{ij}}^{2},i=1,\dots ,N,$$

where ${\lambda }_{j}$ is $j$-th eigenvalue and ${w}_{j}=[{w}_{1j},{w}_{2j},\dots ,{w}_{Nj}]$ is $j$-th eigenvector. According to this metric, the importance of particular input can be expressed through how changes of particular input changes function on average. ASFS interpretation^60^ explains that scaling each eigenvector by its corresponding eigenvalue is reasonable for global sensitivity metric construction. Eigenvector ${w}_{1}$ identifies the most important direction in the parameter space in the following sense: perturbing input along ${w}_{1}$ changes $f$ more, than perturbing input orthogonal to ${w}_{1}$^60^. The components of ${w}_{1}$ measure the relative change in each component of input along this most important direction, so they impart significance to each component of input. The second most important direction is the eigenvector ${w}_{2}$ and relative importance of ${w}_{2}$ is measured by the difference between eigenvalues ${\lambda }_{1}$ and ${\lambda }_{2}$^60^. ASFS (15) are bounded above by the Derivative based global sensitivity metric given by (3)^60^:

16 $${\alpha }_{FSi}\left(M\right)\le {v}_{i}$$

where for $M=N$ inequality becomes equality:

17 $${\alpha }_{FSi}\left(M\right)={v}_{i}$$

### ANOVA overview

ANOVA is a statistical tool for the detection of differences between group means. Partitioning of variance includes calculations of Sum of Squares (SS). SS is divided on between groups SS (${SS}_{b}$) and within groups SS (${SS}_{w}$)^72^:

18 $$SS_{i} = \mathop \sum \limits_{s = 1}^{{N_{s} }} \left( {f_{i} \left( {x_{i}^{s} } \right) - \overline{{f_{i} \left( {x_{i} } \right)}} } \right)^{2} ,\;\;\;1 \le i \le N$$

19 $${SS}_{w}=\sum_{i=1}^{N}{SS}_{i}$$

20 $${SS}_{b}=\sum_{i=1}^{N}{N}_{s}{\left(\overline{f\left({x}_{i}\right)}-\overline{f\left(x\right)}\right)}^{2}$$

where ${N}_{s}$ is the number of samples in the group determined by ${x}_{i}$. Sum of squares for the categorical variable is given by^73^.

21 $${SS}_{i}=\frac{1}{2{N}_{s}}\sum_{{s}_{1}}\sum_{{s}_{2}}{\left({f}_{i}\left({x}_{i}^{{s}_{1}}\right)-{f}_{i}\left({x}_{i}^{{s}_{2}}\right)\right)}^{2}, 1\le i\le N,$$

22 $${f}_{i}\left({x}_{i}^{{s}_{1}}\right)-{f}_{i}\left({x}_{i}^{{s}_{2}}\right)=\left\{\begin{array}{c}1\\ 0\end{array}\right.\begin{array}{c}\mathrm{ if }{f}_{i}\left({x}_{i}^{{s}_{1}}\right)\ne {f}_{i}\left({x}_{i}^{{s}_{2}}\right)\\ \mathrm{ if}{ f}_{i}\left({x}_{i}^{{s}_{1}}\right)={f}_{i}\left({x}_{i}^{{s}_{2}}\right)\end{array},$$

23 $${SS}_{i}=\frac{{N}_{s}}{2}\left(1-\sum_{\delta }{p\left(\delta \right)}^{2}\right), 1\le i\le N,$$

where $p\left(\delta \right)$ is the probability that ${f}_{i}\left({x}_{i}\right)$ takes the value $\delta$.

## Introduction of new method and metrics

According to Fig. 1, in this chapter mathematical background of new methods and metrics is given and appropriate notation is introduced.

### Dimensionality reduction method: modified ASM

In this paper, we propose a modification of ASM where variance/STD is used as a measure of the function variability. STD and variance operate on one-dimensional space and can be calculated for each dimension independently of the other dimensions.

#### Definition 1

*Let the function *$f(x)$* be a well-defined real-valued function on *$N$*-dimensional hypercube, i.e. *$x=\left[{x}_{1},{x}_{2},\dots ,{x}_{N}\right]\in {\left[-\mathrm{1,1}\right]}^{N}$* where all components of *$\mathrm{x}$* are independent. Assume that *${\sigma }^{2}<\infty$* where *${\sigma }^{2}$* is variance of *$f(x)$*. Let input column vector be.*

24 $${STD}_{x}=\left[\begin{array}{c}S(1)\sqrt{{SS}_{1}}\\ \vdots \\ S(N)\sqrt{{SS}_{N}}\end{array}\right]$$

*where *${SS}_{i}$* is given with *(18)* or *(23), $S(i)$* is the function defined as*

25 $$S\left(i\right)=\left\{\begin{array}{c}\begin{array}{cc}-1& \mathrm{if } \, {f}_{i} \, \left({x}_{i}\right) \, is \, descending\end{array}\\ \begin{array}{cc}+1& \mathrm{if }\, {f}_{i} \, \left({x}_{i}\right) \, is \, increasing\end{array}\end{array}\right.$$

*for all *$i=\mathrm{1,2},\dots ,N$*. Covariance matrix C is the outer product of *${STD}_{x}$* with itself; i.e.*

26 $$C=\underset{\chi }{\overset{}{\int }}\left({STD}_{x}\right){\left({STD}_{x}\right)}^{T}\rho (x)dx$$

*where *$\chi \subseteq {\left[-\mathrm{1,1}\right]}^{N}$* is the domain of interest of function *$f$* and *$\rho \left(x\right)$* is uniform probability density function on hypercube, i.e. *$\rho \left(x\right)={2}^{-N}$* for *$x\in {[-\mathrm{1,1}]}^{N}$* and zero elsewhere.*

The eigendecomposition of $C$ given in (6) and (26) yields information about the directions along which function $f(x)$ varies for both ASM and mASM respectively. The properties of interest of matrix $C$ in mASM (26) compared to matrix $C$ in ASM (6), remain the same. Basically, in both cases, the matrices $C$ are real and symmetric, and thus have the factorization $W\Lambda {W}^{T}$ with real eigenvalues in $\Lambda$ and orthonormal eigenvectors in the columns of $W$^74^. Orthogonality property $W{W}^{T}={W}^{T}W=E$ implies ${W}^{T}CW=\Lambda$, thus *i*-th eigenvalue can be represented as

27 $${\lambda }_{i}={{w}_{i}}^{T}C{w}_{i},$$

where ${w}_{i}$ is the corresponding eigenvector.

Definition (26) of $C$ for the mSAM, combined with basic properties of matrix algebra, give the following representation

28 $${\lambda }_{i}=\underset{\chi }{\overset{}{\int }}{\left({{{w}_{i}}^{T}STD}_{x}\right)}^{2}\rho (x)dx.$$

Thus, ${\lambda }_{i}=0$ implies that the function under consideration does not change in the direction of the ${w}_{i}$. Similarly, sufficiently small values of eigenvalues correspond to small changes of function of consideration, analogously as in the case of ASM.

Here, ${SS}_{i}$ is calculated for at most ${N}_{s}$ points in each direction, so the required number of simulations in this case is

29 $${N}_{sim}={\alpha }_{of}M({N}_{s}N+1)\mathit{log}N.$$

**Algorithm 2: mASM**

1. Take ${N}_{p}={\alpha }_{of}M\mathrm{log}N$ independent points $\left\{{x}^{1},{x}^{2},\dots ,{x}^{{N}_{p}}\right\}$ from the domain of interest according to the probability density function $\rho (x)$.
2. For each point ${x}^{p}$, calculate values ${SS}_{i}$ using additional ${N}_{s}$ samples for $\forall i\in \left\{\mathrm{1,2},\dots ,N\right\}$.
3. Form input column vector (24) and approximate:
  30 $$\widehat{C}=\frac{1}{{N}_{p}}\sum_{j=1}^{{N}_{p}}{STD}_{{x}^{p}}{{STD}_{{x}^{p}}}^{T}.$$
4. Compute the eigendecomposition
  31 $$\widehat{C}=\widehat{W}\widehat{\Lambda }{\widehat{W}}^{T}$$
  where $\widehat{\Lambda }=diag({\widehat{\lambda }}_{1},\dots ,{\widehat{\lambda }}_{N})$ is the diagonal matrix of eigenvalues arranged in decreasing order and $\widehat{W}$ is the matrix of eigenvectors ordered accordingly. Analogously as for Algorithm 1.
  32 $$\frac{1}{\sqrt{{N}_{p}}}\left[{\mathrm{STD}}_{{\mathrm{x}}^{1}}\dots {\mathrm{STD}}_{{\mathrm{x}}^{{\mathrm{N}}_{\mathrm{p}}}}\right]=\widehat{W}\sqrt{\widehat{\Lambda }}\widehat{V}$$

### Feature selection metric: variance scores for feature selection

Variance scores for feature selection VSFS is a sensitivity metric introduced analogously to ASFS.

#### Definition 2

*Let *$C$* be defined by * (26),* eigendecomposition of *$C$* be *$C=W\Lambda {W}^{T}$*, where* $\Lambda =diag({\lambda }_{1},\dots ,{\lambda }_{N})$, *and *$W=({w}_{ij})$*. Variance/STD scores for feature selection VSFS for reduction degree *$M$* is*

33 $$\beta _{{FS_{i} }} = \beta _{{FS_{i} }} \left( M \right) = \sum\limits_{{j = 1}}^{M} {\lambda _{j} } w_{{ij}}^{2} ,\;\;i \in \left\{ {1,2, \ldots ,N} \right\}$$

Comparison of ASM and mASM is given in Table 2.

**Table 2:** Comparison of ASM and mASM.

Dimensionality reduction method	ASM	mASM
Input column vector	$${\nabla }_{\mathrm{x}}\mathrm{f}\left(\mathrm{x}\right)=\left[\begin{array}{c}\frac{\partial \mathrm{f}}{\partial {\mathrm{x}}_{1}}(\mathrm{x})\\ \vdots \\ \frac{\partial \mathrm{f}}{\partial {\mathrm{x}}_{\mathrm{N}}}(\mathrm{x})\end{array}\right]$$	$${STD}_{x}=\left[\begin{array}{c}S(1)\sqrt{{SS}_{1}}\\ \vdots \\ S(N)\sqrt{{SS}_{N}}\end{array}\right]$$
Matrix C	$C=\int \left({\nabla }_{x}f\right){\left({\nabla }_{x}f\right)}^{T}\rho (x)dx,$	$C=\int \left({\mathrm{STD}}_{\mathrm{x}}\right){\left({\mathrm{STD}}_{\mathrm{x}}\right)}^{T}\rho (x)dx$
Approximation of matrix C	$\widehat{C}=\frac{1}{{N}_{p}}\sum_{j=1}^{{N}_{p}}{\nabla }_{x}{\mathrm{f}}^{\mathrm{p}}{\nabla }_{x}{{f}^{p}}^{T}$	$\widehat{C}=\frac{1}{{N}_{p}}\sum_{j=1}^{{N}_{p}}{\mathrm{STD}}_{{\mathrm{x}}^{\mathrm{p}}}{{\mathrm{STD}}_{{\mathrm{x}}^{\mathrm{p}}}}^{\mathrm{T}}$
Eigendecomposition of C	$\frac{1}{\sqrt{{N}_{p}}}\left[{\nabla }_{x}{f}^{1}\dots {\nabla }_{x}{\mathrm{f}}^{{\mathrm{N}}_{\mathrm{p}}}\right]=\widehat{W}\sqrt{\widehat{\Lambda }}\widehat{V}$	$\frac{1}{\sqrt{{N}_{p}}}\left[{\mathrm{STD}}_{{\mathrm{x}}^{1}}\dots {\mathrm{STD}}_{{\mathrm{x}}^{{\mathrm{N}}_{\mathrm{p}}}}\right]=\widehat{W}\sqrt{\widehat{\Lambda }}\widehat{V}$
Number of points	${N}_{p}={\alpha }_{of}M\mathrm{log}N$	${N}_{p}={\alpha }_{of}M\mathrm{log}N$
Number of simulations	${N}_{sim}={\alpha }_{of}k(N+1)\mathrm{log}N$	${N}_{sim}={\alpha }_{of}k({N}_{S}N+1)\mathrm{log}N$
Sensitivity metric	${{\alpha }_{FS}}_{i}={\alpha }_{{FS}_{i}}\left(M\right)=\sum\nolimits_{j=1}^{M}{\lambda }_{j}{{w}_{ij}}^{2}$	${{\beta }_{FS}}_{i}={\beta }_{{FS}_{i}}\left(M\right)=\sum\nolimits_{j=1}^{M}{\lambda }_{j}{{w}_{ij}}^{2}$

*ASM* Active subspaces method, *mASM* modified ASM.

### Feature extraction metric: activity scores for feature extraction

A new metric for feature extraction is introduced in order to be able to compare the result for each reduction degree $M\le N$ of feature extraction and feature selection approach. Analogously as in the case of ASFS, scaling each eigenvector by its corresponding eigenvalue is a base for specification of Activity scores for feature extraction ASFE of dimension $M$.

#### Definition 3

*Let *${\lambda }_{j}$* and *${w}_{ij}$* be defined as in* (15),* Activity scores for feature extraction of reduction degree* $M, 1\le M\le N$* is*

34 $${{\alpha }_{FE}}_{M}=\sum_{j=1}^{M}\sum_{i=1}^{N}{\lambda }_{j}{{w}_{ij}}^{2}$$

Important properties of this metric are proven in the following theorems. ASFS ${\alpha }_{FSi}$ for specified $M\le N$ is column vector ${\left\{{\alpha }_{FSi}\right\}}_{i=1}^{N}$ containing information about the importance of all inputs $i$. ASFE ${{\alpha }_{FE}}_{M}$ for specified $M\le N$ is a scalar containing information about the importance of the linear combination of all inputs $i$.

#### Theorem 1

*The Activity scores for feature extraction* ${{\alpha }_{FE}}_{M}$ *, correspond to sum of eigenvalues* ${\left\{{\lambda }_{i}\right\}}_{i=1}^{M}$ *:*

35 $${{\alpha }_{FE}}_{M}=\sum_{j=1}^{M}\sum_{i=1}^{N}{\lambda }_{j}{{w}_{ij}}^{2}=\sum_{j=1}^{M}{\lambda }_{j}$$

#### Proof

${{\alpha }_{FE}}_{M}=\sum_{j=1}^{M}\sum_{i=1}^{N}{\lambda }_{j}{{w}_{ij}}^{2}=\sum_{j=1}^{M}{\lambda }_{j}\sum_{i=1}^{N}{{w}_{ij}}^{2}$. Since $W$ is an orthogonal matrix whose columns are normalized eigenvectors, $\sum_{i=1}^{N}{{w}_{ij}}^{2}=1$, so ${{\alpha }_{FE}}_{M}=\sum_{j=1}^{M}{\lambda }_{j},$ as required.

#### Theorem 2

${{\alpha }_{FE}}_{M}$* is always greater than or equal to* ${{\alpha }_{FS}}_{i}(N)$,* for* $i=1,\dots ,N$,* where* $1\le M\le N$:

36 $${{\alpha }_{FE}}_{M}\ge {{\alpha }_{FS}}_{i}(N)$$

#### Proof

For $M=1$ and selected input ${IF}_{s}$, using characteristics of matrix W: $W{W}^{^{\prime}}=I$ and $W{^{\prime}}W=I$ result can be derived. (36) reduces to:

$${{{\alpha }_{FE}}_{1}=\lambda }_{1}\ge \sum_{j=1}^{N}{{\lambda }_{j}{w}_{sj}}^{2}={{\alpha }_{FS}}_{s}(N),$$

which can be written equivalently as.

$${\lambda }_{1}\ge {\lambda }_{1}{{w}_{s1}}^{2}+{\lambda }_{1}\sum_{j=2}^{N}{{w}_{sj}}^{2}-{\lambda }_{1}\sum_{j=2}^{N}{{w}_{sj}}^{2}+\sum_{j=2}^{N}{{{\lambda }_{j}w}_{sj}}^{2},$$

$${\lambda }_{1}\ge {\lambda }_{1}\sum_{j=1}^{N}{{w}_{sj}}^{2}+\sum_{j=2}^{N}({{{\lambda }_{j}-{\lambda }_{1})w}_{sj}}^{2},$$

$$\sum_{j=2}^{N}({{{\lambda }_{j}-{\lambda }_{1})w}_{sj}}^{2}\le {\lambda }_{1}-{\lambda }_{1}\sum_{j=1}^{N}{{w}_{sj}}^{2},$$

$$\mathrm{i}.\mathrm{e}. \sum_{j=2}^{N}({{{\lambda }_{j}-{\lambda }_{1})w}_{sj}}^{2}\le 0.$$

Since ${\lambda }_{j}\le {\lambda }_{1}$ for all $j=2,\dots ,N$ last inequality holds true, i.e. ${{\alpha }_{FE}}_{M}\ge {{\alpha }_{FS}}_{i}$ for all $i=1,\dots ,N$ as required. For $M\ge 2$ and selected set of inputs $\left\{{x}_{i}\right\},$ where $i\in {S}_{i}$, and ${S}_{i}$ is set of selected inputs, $\left|{S}_{i}\right|=M$, using characteristics of matrix $W$: $W{W}^{^{\prime}}=I$ and $W{^{\prime}}W=I$, proof can be derived:

$$\sum_{j=1}^{M}{\lambda }_{j}\ge \sum_{j=1}^{N}\sum_{i\in {S}_{i}}{{\lambda }_{j}{w}_{ij}}^{2},$$

so, the claim can be written in the form.

$$\sum_{j=1}^{M}{\lambda }_{j}\ge {\lambda }_{1}\sum_{i\in {S}_{i}}{{w}_{i1}}^{2}+{\lambda }_{1}\sum_{j=2}^{N}\sum_{i\in {S}_{i}}{{w}_{ij}}^{2}-{\lambda }_{1}\sum_{j=2}^{N}\sum_{i\in {S}_{i}}{{w}_{ij}}^{2}+\sum_{j=2}^{N}\sum_{i\in {S}_{i}}{{\lambda }_{j}{w}_{ij}}^{2},$$

$$\sum_{j=1}^{M}{\lambda }_{j}\ge {M\lambda }_{1}+\sum_{j=2}^{N}\sum_{i\in {S}_{i}}{{(\lambda }_{j}-{\lambda }_{1}){w}_{ij}}^{2},$$

i.e. we need to prove.

$${M\lambda }_{1}-\sum_{j=1}^{M}{\lambda }_{j}+\sum_{j=2}^{N}\sum_{i\in {S}_{i}}{{(\lambda }_{j}-{\lambda }_{1}){w}_{ij}}^{2}\le 0,$$

or equivalently.

$${M\lambda }_{1}-\sum_{j=1}^{M}{\lambda }_{j}+\sum_{j=2}^{N}{(\lambda }_{j}-{\lambda }_{j-1})\sum_{k=j}^{N}\sum_{i\in {S}_{i}}{{w}_{ik}}^{2}\le 0,$$

$$-\sum_{j=2}^{M}{(\lambda }_{j}-{\lambda }_{1})+\sum_{j=2}^{N}{(\lambda }_{j}-{\lambda }_{j-1})\sum_{k=j}^{N}\sum_{i\in {S}_{i}}{{w}_{ik}}^{2}\le 0,$$

$$-\sum_{j=2}^{M}{(\lambda }_{j}-{\lambda }_{1})+\sum_{j=2}^{M}{(\lambda }_{j}-{\lambda }_{j-1})\sum_{k=j}^{N}\sum_{i\in {S}_{i}}{{w}_{ik}}^{2}+\sum_{j=M+1}^{N}{(\lambda }_{j}-{\lambda }_{j-1})\sum_{k=j}^{N}\sum_{i\in {S}_{i}}{{w}_{ik}}^{2}\le 0,$$

Since ${\lambda }_{j}-{\lambda }_{j-1}\le 0$ directly follows that.

$$\sum_{j=M+1}^{N}{(\lambda }_{j}-{\lambda }_{j-1})\sum_{k=j}^{N}\sum_{i\in {S}_{i}}{{w}_{ik}}^{2}\le 0,$$

so, it is left to show.

$$-\sum_{j=2}^{M}{(\lambda }_{j}-{\lambda }_{1})+\sum_{j=2}^{M}{(\lambda }_{j}-{\lambda }_{j-1})\sum_{k=j}^{N}\sum_{i\in {S}_{i}}{{w}_{ik}}^{2}\le 0,$$

$$-\sum_{j=2}^{M}{(\lambda }_{j}-{\lambda }_{1})+\sum_{j=2}^{M}{(\lambda }_{j}-{\lambda }_{j-1})\sum_{i\in {S}_{i}}\left(1-\sum_{k=1}^{j-1}{{w}_{ik}}^{2}\right)\le 0,$$

$$-\sum_{j=2}^{M}{(M-j+1)(\lambda }_{j}-{\lambda }_{j-1})+\sum_{j=2}^{M}{(\lambda }_{j}-{\lambda }_{j-1})\sum_{i\in {S}_{i}}\left(1-\sum_{k=1}^{j-1}{{w}_{ik}}^{2}\right)\le 0,$$

$$\sum_{j=2}^{M}{(\lambda }_{j}-{\lambda }_{j-1})\left(\left(-M+j-1\right)+\sum_{i\in {S}_{i}}1-\sum_{i\in {S}_{i}}\sum_{k=1}^{j-1}{{w}_{ik}}^{2}\right)\le 0,$$

$$\sum_{j=2}^{M}{(\lambda }_{j}-{\lambda }_{j-1})\left(j-1-\sum_{i\in {S}_{i}}\sum_{k=1}^{j-1}{{w}_{ik}}^{2}\right)\le 0,$$

$$\sum_{j=2}^{M}{(\lambda }_{j}-{\lambda }_{j-1})\left(\sum_{k=1}^{j-1}1-\sum_{k=1}^{j-1}\sum_{i\in {S}_{i}}{{w}_{ik}}^{2}\right)\le 0,$$

$$\sum_{j=2}^{M}{(\lambda }_{j}-{\lambda }_{j-1})\left(\sum_{k=1}^{j-1}\left(1-\sum_{i\in {S}_{i}}{{w}_{ik}}^{2}\right)\right)\le 0,$$

$$\sum_{j=2}^{M}{(\lambda }_{j}-{\lambda }_{j-1})\left(\sum_{k=1}^{j-1}\sum_{i\notin {S}_{i}}{{w}_{ik}}^{2}\right)\le 0,$$

Since ${\lambda }_{j}\le {\lambda }_{j-1}$ for all $j=2,\dots ,N$, last inequality holds true, i.e.${{\alpha }_{FE}}_{M}\ge {{\alpha }_{FS}}_{i}(N)$ for all $i=1,\dots ,N$ and for all $M$ as required. The proof is completed.

According to (36) it can be concluded that in QoE modeling linear transformation of the input set of IFs by weighted coefficients determined by ASM, reduced space specified by ${IF}_{new}$ will always contain the same or more information about changes of QoE than the selection of any set of input IFs.

#### Theorem 3

*Activity scores for feature extraction for* $M=N$ *is equal to the sum of all* $i=1,\dots ,N$ *Activity scores for feature selection for* $M=N$:

37 $${{\alpha }_{FE}}_{N}=\sum_{i=1}^{N}{\alpha }_{{FS}_{i}}\left(N\right).$$

#### Proof

$${{\alpha }_{FE}}_{N}=\sum_{i=1}^{N}{\alpha }_{{FS}_{i}}\left(N\right)=\sum_{j=1}^{N}\sum_{i=1}^{N}{\lambda }_{j}{{w}_{ij}}^{2}=\sum_{i=1}^{N}\sum_{j=1}^{N}{\lambda }_{j}{{w}_{ij}}^{2},$$

as required.

Using properties proven in Theorem 3 it is possible to specify the relative ratio which expresses a ratio between function variability determined by selected reduction degree and selected approach, and cumulative function variability described by (37).

#### Definition 4

*Let the *${{\alpha }_{FE}}_{M}$* and *${\alpha }_{{FS}_{i}}\left(M\right)$* be defined as in* (34)* and* (15)* respectively, relative ratio for feature extraction is*

38 $$R\left(\%\right)=\frac{{{\alpha }_{FE}}_{M}}{{{\alpha }_{FE}}_{N}}\cdot 100$$

*whereas for feature selection is*

39 $$R\left(\%\right)=\frac{{\alpha }_{{FS}_{i}}\left(N\right)}{\sum_{i=1}^{N}{\alpha }_{{FS}_{i}}\left(N\right)}\cdot 100$$

### Feature extraction metric: variance/STD scores for feature extraction

Analogously to ASFE, Variance/STD scores for feature extraction VSFE can be defined as:

#### Definition 5

*Let the *${\lambda }_{j}$* and *${w}_{ij}$* be defined as in* (33),* Variance/STD scores for feature extraction VSFE is*

40 $${{\beta }_{FE}}_{M}=\sum_{j=1}^{M}\sum_{i=1}^{N}{\lambda }_{j}{{w}_{ij}}^{2},i=1,\dots ,M,\dots ,N$$

*VSFE retains all proofed performance as ASFE*.

## Numerical analysis

Based on the above-mentioned mathematical introduction of method and metrics, in this chapter numerical analysis is performed which includes multiple simulations in order to obtain QoE for IFs analysis. The following tools are used. MATLAB^75^ is used as a tool for random selection of values of input IFs, implementation of the ASM and mASM, calculation of metrics, neural network modelling, and data analysis. The video sequence is coded using the ffmpeg tool^76^, video transmission simulation is performed in the NS3 simulator^77^ using the EvalVid evaluation tool^78^ for QoE metric estimation. The video sequence is widely used sequence Akiyo (352 × 288 resolution with 30 fps each 10 s long) and can be accessed from^79^. Objective measurement of the QoE is made for selected input points. MOS tool is based on MOS calculation of every single frame of the received video and its comparison to the MOS of every single frame of the original video. MOS determination for all input points, approximated gradients and samples is used as input to form input column vector for ASM and mASM. In this paper, two sets of IFs are analysed. Set 1 includes IFs to which both ASM and mASM methods can be applied. Set 2 includes IFs from Set 1 and an additional IF which is a categorical variable. For Set 2, the mASM method is applied since the calculation of gradients for categorical variables is not possible and therefore the application of ASM method is not possible. Overview of the input IFs is given in Table 3.

**Table 3:** Overview of the input IFs.

			Input IFs	Range	Step
IFs set 2	IFs set 1	IF_1_	Distance	$d$: 1–151 [m]	$\Delta d$: 6 [m]
		IF_2_	Buffer size	$BS:$ 1–76 [packets]	$\Delta BS$: 3 [packets]
		IF_3_	Packet size	$PS:$ 512–2112 [byte]	$\Delta PS$: 64 [byte]
		IF_4_	Fragment size	$FS:$ 256–2431 [byte]	$\Delta FS$: 87 [byte]
		IF_5_	RTS/CTS threshold	$R/C:$ 0–2325 [byte]	$\Delta R/C$: 93 [byte]
		IF_6_	Constant rate factor	$CRF$:0–50 [comp. ratio]	$\Delta CRF$: 2 [comp. ratio]
		IF_7_	Frames per second	$FPS:$ 5–30 [fps]	$\Delta FPS$: 1 [fps]
		IF_8_	Max Ssrc	$Ssrc$: 0–25 [no]	$\Delta Ssrc$: 1 [no]
		IF_9_	Max Slrc	$Slrc$: 0–25 [no]	$\Delta Slrc:$ 1 [no]
		IF_10_	Rx noise figure	$RxN$: 0–2 [dB]	$\Delta RxN:$ 0.08 [dB]
		IF_11_	Physical mode	DsssRate1Mbps DsssRate2Mbps DsssRate5_5Mbps DsssRate11Mbps	

*RTS* Request to Send, *CTS* Clear to Send.

### Eigenvalues evaluation, ASFS/VSFS estimation, and performance analysis

**Figure 3:**
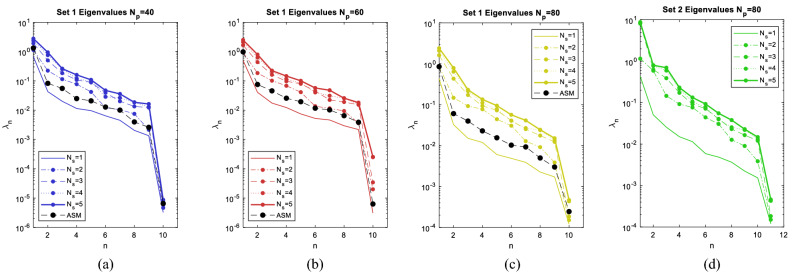
Eigenvalues approximation for (**a**) Set 1 for ${N}_{p}=40$, (**b**) Set 1 for ${N}_{p}=60$, (**c**) Set 1 for ${N}_{p}=80$ (**d**) Set 2 for ${N}_{p}=80$.

Selected values of input IFs uniquely determine 10-dimensional point as input for Set 1 and 11-dimensional point as input for Set 2. Input points are randomly selected by random selection of all input IFs. The required number of points is determined according to (5), so simulation results are obtained for 40/60/80 input points. A comparison of ASM and mASM methods is performed for Set 1. The results of applying the mASM method to Set 2 are also given below. According to ASM and mASM procedure, input column vector of gradients (see (7)) is used to approximate eigenvalues and eigenvectors for ASM, whereas input column vector of variances [see (24)] is used in case of mASM (Fig. 3).

Figure 3c shows a gap between ${\widehat{\lambda }}_{1}$ and ${\widehat{\lambda }}_{2}$ implying possibility to reduce dimension to one for both ASM and all ${N}_{s}$ for mASM for ${N}_{p}=80$. Gap in eigenvalues indicates the separation between the active and inactive subspace, and computed eigenvectors are more accurate when there is a significant gap between eigenvalues. The values of eigenvalues show that the change in function in 10th dimension is negligible. Comparing ASM and mASM, different gaps in these methods are the result of different measures of function variability. Although the larger number of input points gives better accuracy of the prediction, a similar gap explanation can be done for eigenvalues for 40/60 input points (Fig. 3a,b). Figure 3d gives an overview of eigenvalues for Set 2 for 11 IFs only for mASM, where the separation between active and inactive subspace can also be observed.

**Figure 4:**
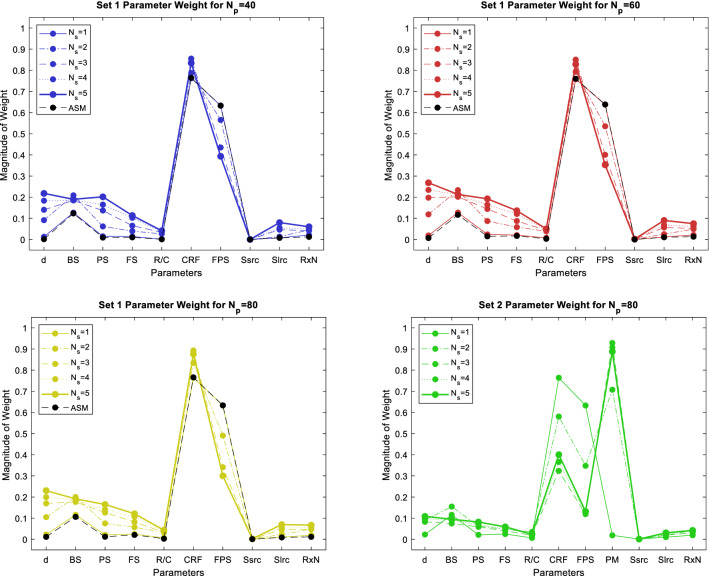
Magnitude of the components of approximated first eigenvector (**a**) Set 1 for ${N}_{p}=40$, (**b**) Set 1 for ${N}_{p}=60$, (**c**) Set 1 for ${N}_{p}=80$ (**d**) Set 2 for ${N}_{p}=80$.

Dominant changes exist in the first dimension. The magnitude of the components of the approximated eigenvector corresponding to ${\widehat{\lambda }}_{1}$ is given in Fig. 4. For ASM and mASM, weighted coefficients for ${N}_{p}=40$ have approximately the same values as for ${N}_{p}=60$ and ${N}_{p}=80$, so it is not expected that further increase in the number of points will lead to significant changes in values of weighted coefficients. For Set 2, it is important to note that the addition of a new IF leads to new weighted coefficients, whose values depend on the particular IF influence on the QoE metric.

**Figure 5:**
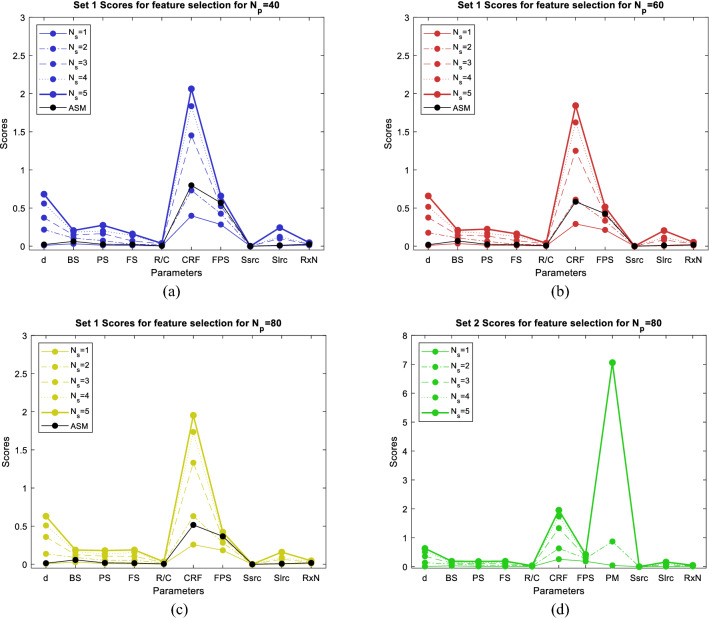
Activity scores for feature selection ASFS and Variance/STD scores for feature selection VSFS for ${N}_{s}=\left\{\mathrm{1,2},\mathrm{3,4},5\right\}$ (**a**) Set 1 for ${N}_{p}=40$, (**b**) Set 1 for ${N}_{p}=60$, (**c**) Set 1 for ${N}_{p}=80$ (**d**) Set 2 for ${N}_{p}=80$.

ASFS ${\alpha }_{FS}$ and VSFS ${\beta }_{FS}$ are used to determine importance of a model input IFs [see (15) and (33)]. Figure 5a–c present ASFS and VSFS for all input IFs. Similar results are obtained for different number of input points. According to ASFS and VSFS, the dominant IF is CRF. After CRF, the next important IF is FPS for ASM, and distance for mASM. It can be concluded that small variation in these IFs, changes the QoE metric more than the small variation of other IFs. Accuracy of the prediction increases with an increase of input points so sensitivity analysis given for ${N}_{p}=80$ will be used for comparison with global metrics. Comparison of ASFS and VSFS for ${N}_{p}=80$ and for ${N}_{s}=5$ (VSFS) gives a different order of importance. For both methods the tenth parameter is MaxSsrc with the least influence on QoE metric. The different order is due to different metrics that measure the function variability and also the observed different order is also due to the fact that the parameters have approximately the same effect on the QoE metric. For Set 2, the dominant IF is PM, followed by CRF, d, FPS, FS, BS, PS, MaxSlrc, RxNoise, R/C, and MaxSsrc. By ignoring categorical IFs, the most influential IF is neglected, confirming that as many IFs as possible need to be considered for an accurate QoE estimate.

The accuracy of dimensionality reduction methods is tested using additional simulation measurements. In this phase, data modelling is done to a rotated and reduced set of input IFs using Neural Network according to^80^. According to^81^, performance evaluation is done using the following evaluation indexes:

- Pearson Correlation Coefficient (PCC)
  41 $$PCC=\frac{\sum_{i=1}^{N}\left({QoE}_{i}-\overline{QoE }\right)\left({\widehat{QoE}}_{i}-\overline{\widehat{QoE} }\right)}{\sqrt{\sum_{i=1}^{N}{\left({QoE}_{i}-\overline{QoE }\right)}^{2}}\sqrt{\sum_{i=1}^{N}{\left(\widehat{{QoE}_{i}}-\overline{\widehat{QoE} }\right)}^{2}}}$$
- Root Mean Square Error (RMSE)
  42 $$RMSE=\sqrt{\frac{1}{N}\sum_{i=1}^{N}{\left({QoE}_{i}-\widehat{{QoE}_{i}}\right)}^{2}}$$
- Mean Absolute Error (MAE)
  43 $$MAE=\frac{1}{N}\sum_{i=1}^{N}\left|{QoE}_{i}-\widehat{{QoE}_{i}}\right|$$
- And Root relative squared error (RRSE)
  44 $$RRSE=\sqrt{\frac{\sum_{i=1}^{N}{\left({QoE}_{i}-\widehat{{QoE}_{i}}\right)}^{2}}{\sum_{i=1}^{N}{\left({QoE}_{i}-\overline{QoE }\right)}^{2}}}$$
  where ${QoE}_{i}$ is obtained QoE metric, ${\widehat{QoE}}_{i}$ is estimated QoE metric, and $N$ is number of the observation.

**Figure 6:**
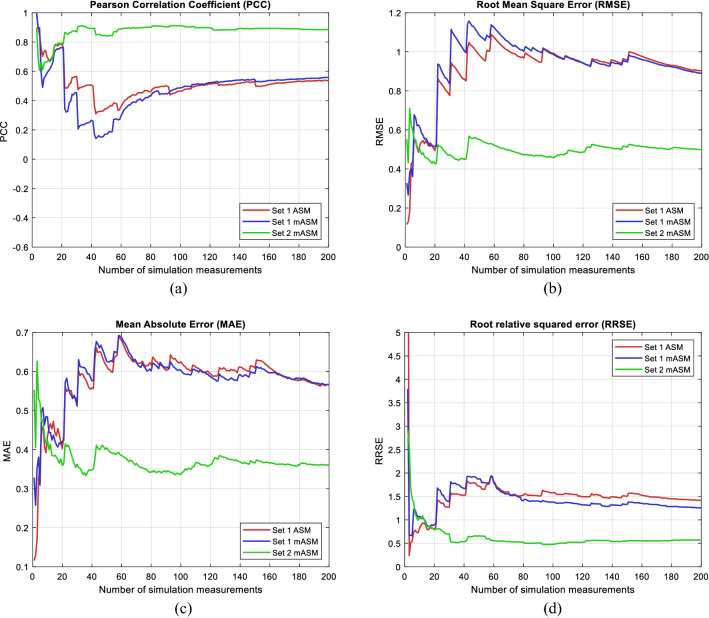
Performance analysis: (**a**) Pearson Correlation Coefficient (PCC), (**b**) Root Mean Square Error (RMSE), (**c**) Mean Absolute Error (MAE) (**d**) Root relative squared error (RRSE).

In Fig. 6 it can be seen that the accuracy of mASM methods with 11 IFs is significantly higher than mASM with 10 IFs and ASM with 10 IFs. Adding new IFs in QoE estimation increases prediction accuracy. The accuracy of prediction for the ASM and mASM method with 10 IFs is approximately the same, and through the accuracy of prediction it can be seen once again that neglecting the most important IF significantly reduces the accuracy of prediction.

### Comparison with global metrics

Comparison of ASFS and VSFS with global metrics is done for Set 1 where ${N}_{p}=80$ is used for ASFS and it is compared to Derivative based global sensitivity metric [see (4)]. Sensitivity analysis given for ${N}_{p}=80$ and ${N}_{s}=1$ for VSFS is compared with Sobol’s total sensitivity metric [see (2)] where ${N}_{s}=1$. The same samples are used for the calculation of VSFS and Sobol’s total sensitivity metric. According to (17), ASFS is equal to Derivative based global sensitivity metric for $M=N$ as can be seen in Table 4. Analogously to ASFS, VSFS is equal to Sobol’s total sensitivity metric multiplied by approximated variance ${{\widehat{\sigma }}_{f}}^{2}$ for $M=N$ (Table 5). It can be concluded that besides the fact that ASFS is consistent with rankings produced by Derivative based global sensitivity metrics^60^, VSFS is also consistent with rankings produced by Sobol’s total sensitivity metric. For nicely behaved functions all metrics are consistent which is a common case for practical models. As we have shown, this is the case in our example for QoE metric where the most important IFs are recognized as the most important for all metrics as well as the unimportant IFs.

**Table 4:** Comparison of activity scores for feature selection and derivative based global sensitivity metric.

	Activity scores for feature selection ${{\alpha }_{FS}}_{i}$										Derivative based metric ${v}_{i}$
	M = 1	M = 2	M = 3	M = 4	M = 5	M = 6	M = 7	M = 8	M = 9	M = N = 10	
${IF}_{1}$	0.0001	0.0001	0.0013	0.0013	0.0118	0.0133	0.0143	0.0144	0.0145	0.0145	0.0145
${IF}_{2}$	0.0096	0.0501	0.0505	0.0566	0.0566	0.0570	0.0570	0.0570	0.0570	0.0570	0.0570
${IF}_{3}$	0.0001	0.0068	0.0095	0.0120	0.0123	0.0160	0.0183	0.0187	0.0187	0.0187	0.0187
${IF}_{4}$	0.0004	0.0039	0.0041	0.0076	0.0095	0.0095	0.0150	0.0151	0.0152	0.0152	0.0152
${IF}_{5}$	0	0.0001	0.0005	0.0011	0.0017	0.0017	0.0018	0.0044	0.0056	0.0056	0.0056
${IF}_{6}$	0.5004	0.5009	0.5150	0.5158	0.5159	0.5160	0.5160	0.5160	0.5160	0.5160	0.5160
${IF}_{7}$	0.3423	0.3467	0.3655	0.3661	0.3664	0.3664	0.3665	0.3665	0.3665	0.3665	0.3665
${IF}_{8}$	0	0	0	0	0	0	0	0	0	0.0003	0.0003
${IF}_{9}$	0.0001	0.0031	0.0031	0.0038	0.0043	0.0044	0.0045	0.0063	0.0078	0.0078	0.0078
${IF}_{10}$	0.0001	0.0023	0.0042	0.0124	0.0137	0.0181	0.0183	0.0184	0.0184	0.0184	0.0184

**Table 5:** Comparison of variance/STD scores for feature selection and Sobol’s total sensitivity metric.

	Variance/STD scores for feature selection ${\beta }_{FS}$										Sobol’s total sensitivity metric ${\tau }_{i}$	${\tau }_{i}\cdot {{\widehat{\sigma }}_{f}}^{2}$
	M = 1	M = 2	M = 3	M = 4	M = 5	M = 6	M = 7	M = 8	M = 9	M = N = 10		
${IF}_{1}$	0.0002	0.0016	0.0025	0.0032	0.0040	0.0070	0.0071	0.0072	0.0072	0.0072	0.0153	0.0072
${IF}_{2}$	0.0058	0.0234	0.0235	0.0283	0.0285	0.0285	0.0285	0.0285	0.0285	0.0285	0.0602	0.0285
${IF}_{3}$	0.0002	0.0041	0.0055	0.0062	0.0074	0.0074	0.0092	0.0094	0.0094	0.0094	0.0198	0.0094
${IF}_{4}$	0.0003	0.0028	0.0032	0.0040	0.0058	0.0058	0.0074	0.0074	0.0076	0.0076	0.0161	0.0076
${IF}_{5}$	0	0.0003	0.0004	0.0005	0.0007	0.0007	0.0010	0.0024	0.0028	0.0028	0.0059	0.0028
${IF}_{6}$	0.2519	0.2522	0.2569	0.2580	0.2580	0.2580	0.2580	0.2580	0.2580	0.2580	0.5448	0.2580
${IF}_{7}$	0.1729	0.1759	0.1822	0.1832	0.1832	0.1832	0.1832	0.1832	0.1832	0.1832	0.3869	0.1832
${IF}_{8}$	0	0	0	0	0	0	0	0	0	0.0001	0.0003	0.0001
${IF}_{9}$	0	0.0017	0.0017	0.0019	0.0020	0.0023	0.0023	0.0029	0.0039	0.0039	0.0082	0.0039
${IF}_{10}$	0.0002	0.0021	0.0032	0.0058	0.0076	0.0091	0.0092	0.0092	0.0092	0.0092	0.0195	0.0092

### Comparison of feature extraction and feature selection approaches

In Table 6 Activity scores for all ${\alpha }_{FE}$ are compared with largest ${\alpha }_{FS}$ for Set 1 with the indicated percentage of how much variability QoE metric is described for the considered reduction degree. It can be concluded that feature extracted in the first dimension describes 83.6% of the change of QoE metric [see (38)], whereas the preferred IF for feature selection is CRF which describes 50.6% change of QoE metric [see (39)]. Thus, by introducing a new variable as a linear combination of all input IFs, a one-dimensional space is obtained which describes the largest QoE metric fluctuations, and ignoring other dimensions means ignoring smaller QoE metric oscillations. In contrast, if we choose CRF, all variations of the QoE metric caused by the change of other IFs will be ignored thus neglecting larger QoE metric oscillations. For $M=N=10$ only rotation of initial space is done, so there is no loss and both metrics show that 100% of function variability is described. Selection of type of dimensionality reduction feature selection or feature extraction is always in a favour of feature extraction. If the difference is negligibly small for the selected reduction degree, due to less budgetary complexity, feature selection can be considered as a better choice which would mean that there is a dominant input IF/IFs and the others are negligible or their variations do not change QoE metric at all. In Table 7 Variance/STD scores for all ${\beta }_{FE}$ are compared with largest ${\beta }_{FS}$. Analogously to ASM, Variance/STD scores confirm that the change in QoE metric for the same reduction degree is better described by the linear combination of all IFs compared to the selection of any set of input IFs. A linear combination of input IFs can give a better overview of the change of QoE metric for both the Activity scores and the Variance scores.

**Table 6:** Comparison of activity scores for feature extraction and activity scores for feature selection.

	$M=1$	$M=2$	$M=3$	$M=4$	$M=5$	$M=6$	$M=7$	$M=8$	$M=9$	$M=10$
${\alpha }_{FE}$	0.8532	0.9139	0.9537	0.9766	0.9922	1.0024	1.0117	1.0167	1.0197	1.0200
$R(\%)$	83.6	89.6	93.5	95.7	97.3	98.3	99.2	99.7	99.9	100
${\alpha }_{FS}$	0.5160	0.8825	0.9395	0.9582	0.9767	0.9919	1.0063	1.0141	1.0197	1.0200
$R(\%)$	50,6	86,5	92,1	93,9	95,7	97,2	98,7	99,4	99,9	100

**Table 7:** Comparison of variance/STD scores for feature extraction and variance scores for feature selection.

	$M=1$	$M=2$	$M=3$	$M=4$	$M=5$	$M=6$	$M=7$	$M=8$	$M=9$	$M=10$
${\alpha }_{FE}$	0.4315	0.4640	0.4791	0.4911	0.4971	0.5020	0.5059	0.5081	0.5099	0.5100
$R(\%)$	84.6	91.0	93.9	96.3	97.5	98.4	99.2	99.6	99.9	100
${\alpha }_{FS}$	0.258	0.4412	0.4697	0.4791	0.4883	0.4959	0.5032	0.5071	0.5099	0.5100
$R(\%)$	50.6	86.5	92.1	93.9	95.7	97.2	98.6	99.4	99.9	100

## Discussion

Multidimensional QoE analysis has become imperative to improve the QoE modeling process. The curse of dimensionality is a term that refers to all problems connected with high dimension of data which are surpassed at lower dimensions. High-dimensional data set may contain many features that are all measurements of the same underlying cause, so they are closely related where the features of such data set contain much overlapping information^82^. So according to the posed challenge of achieving dimensionality reduction of the input set of independent IFs in QoE modeling, this study proposes ASM based on derivatives and introduces modification of ASM based on variance/STD as a measure of function variability. The appropriate choice of measure of variability depends on the application. The advantage of mASM in QoE modeling is the possibility to use categorical variables with no need for calculating gradients which is difficult or inadequate for some IFs. Since functional dependency between input IFs and QoE is mostly not known, the use of finite difference methods allows approximation of gradients which introduces an error at the input to the method as opposed to the use of a variance. We also observed that the QoE function mostly does not change much at smaller shifts by dimensions resulting in gradients having a value of 0 in all dimensions. This information is interpreted in the method as all inputs are equally important, although changing a specific input may not change the QoE at all. This results in less important inputs having higher weighted coefficients which is also an additional disadvantage when using gradients. The disadvantage of using variance is the need for more simulations for the calculation. Besides the modification of the existing method, this paper introduces new metrics for the comparison of feature selection and feature extraction approaches.

Application of dimensionality reduction before QoE prediction can provide models for devices with different processors and memory power with varying degrees of complexity and accuracy. Reducing the dimensionality of the input data set can speed up the training process of machine learning algorithms used for QoE prediction. The use of machine learning and large amounts of data in QoE assessment is part of the strategy for developing big data-driven intelligent networks. The development of AI is based on machine learning of big data collected through multiple spots in current and future networks who need to intelligently adjust to the environment, while maintaining quality at a satisfactory level. The estimated QoE can be used as an input to achieve spectral efficiency, energy efficiency, cost reduction, etc. Variations in the quantity of interest using ASM and mASM as dimensionality reduction methods can link resources in the network for optimal resource reservation and architecture design with delay as the quantity of interest and a measure of quality for latency modeling purposes. Quantity of interest could also be power consumption, which is particularly important in implementing solutions that include limited battery life, such as sensors in the IoT networks.

Obtained knowledge in this study can help interested stakeholders including mobile network operators, technology developers, software solution providers and the research community to improve QoE input data by inclusion data preparation phase in order to achieve optimal trade-off between complexity and accuracy, thus optimizing the overall process depends on the specific application. Mobile network operators experience increasing user requirements in the context of quality, which becomes a challenge with the ever-increasing demands of multimedia applications with limited resources. Innovation processes and end user roles are strongly connected, so technology developers cannot simply separate user experience with technology. Optimized QoE inputs that have varying degrees of complexity offer the possibility of applicability across different technologies and prediction models. Software solution providers can improve their algorithms design to meet QoE requirements with appropriate QoE inputs. Academic and research communities can use knowledge to further improve dimensionality reduction methods and QoE prediction models.

This study has several contributions and implications. Firstly, the original contribution of this study is the first attempt, to our best knowledge, to overview the previous applications of dimensionality reduction to the input set of IFs in QoE modeling. This overview contains the applied methods for dimensionality reduction with the achieved degree of dimensionality reduction. This can serve as a basis for ideas to introduce new dimensionality reduction techniques in combination with different algorithms for QoE prediction for different purposes.

Secondly, ASM and mASM as dimensionality reduction techniques used in this study differ in preconditions and outputs from applied dimensionality reduction techniques listed in the overview of related works. A special contribution of the paper is the modification of the ASM method which is not limited to use only in QoE modeling but can be used to reduce the dimensionality of input spaces where categorical or discrete variables are used, and for relatively flat functions. Since the IF input space and the QoE function satisfy both conditions, the preferred method for dimensionality reduction of the independent set of input IFs is mASM. Thirdly, the metrics used and introduced in the paper enable the comparison of different approaches and provide information on QoE changes for different spaces, which is the addition in to the analysis of multidimensional QoE. Understanding the dataset with its strengths and weaknesses is crucial for good QoE prediction since each model learns differently, and what is an advantage in one model may be the weakness of another. Simpler models are generally easier to control and it is possible to more easily identify the reasons for inaccuracies, where the metrics introduced in the paper provide information on the loss of accuracy as a consequence of model simplification.

This study also provides a couple of implications. There are important theoretical implications that show that usage of dimensionality reduction is justified in the QoE preparation phase. Our findings extend the previous work with new method applied on different input set with additional outputs, thus complementing and opening up new possibilities for the application of dimensionality reduction techniques in QoE preparation phase. Practical implications of ASM and mASM usage as dimensionality reduction methods are connected with the requisite for manipulating multiple independent inputs and knowing the change of QoE for variations of all independent inputs. Beside huge volume, data generated within the network also have non-homogenous structure, where input information is incomplete and ambiguous. In addition to the theoretical and practical implications, this study also reveals implications for future research. As the study is limited to 11 input IFs, a future study should extend the above set to give a better picture of the impact of different SIFs, as well as CIFs and HIFs on QoE especially for mASM. Comparison of different QoE prediction techniques in combination with different dimensionality reduction methods would allow a more comprehensive review and performance of the various prediction models will be the focus of future work. In addition, the application of ASM and mASM is not limited to QoE as the quantity of interest so, for example, latency and/or power consumption can be the focus of future work.

## Conclusion

The comprehensive understanding of QoE change requires the analysis of as many IFs as possible which dictates the introduction of tools that can handle spaces with large dimensions and large amounts of data. This is the motivation for this study whose objective is the optimal description of IFs input space depending on the change of QoE. In this regard, a review of related works was made regarding the used dimensionality reduction techniques and an overview of existing techniques suitable for an independent set of input IFs. Modification of method and new metrics are introduced for more comprehensive analysis of IFs and QoE space. The optimal dimensionality reduction approach is feature extraction whereas the optimal reduction degree is the trade-off between the accuracy and complexity.

According to above mentioned, the original contribution of this study follows the objectives and can be summarized as follow:

- Meta-analytical overview of used dimensionality reduction techniques applied to the input set of IFs thus creating a basis for extending the methods used in QoE modeling. LDA, LVQ, and ASM are recognized as methods applicable to an independent set of input IFs, where the advantage of ASM is that it is not limited to classification only;
- Introduction of modification of ASM with variance/STD as a measure of function variability thus overcoming the problem of gradient calculation and creating the possibility of applying the method to discrete and categorical IFs. Modified ASM is not limited to application in QoE modeling;
- Introduction of new metrics ASFE, VSFS, VSFE, and relative ratio R(%) which allows comparison of feature selection and feature extraction approach. It is proved that linear combination of input IFs where weighted coefficients are determined using ASM or mASM method is always a better choice than the selection of any IF or any combination of IFs for given reduction degree;
- Application of ASM and mASM on the selected set of input IFs with an objective evaluation of QoE with the comparison of ASFS and VSFS with global metrics, and comparison of ASFS and ASFE, then VSFS and VSFE metrics for different reduction degrees. Numerical analysis for selected IFs showed that the QoE function is suitable for dimensionality reduction with arbitrary flat directions. It has also been observed that smaller shifts in IFs do not alter QoE much which is another advantage of introducing variance/STD as a measure of function variability in mASM. Performance analysis has shown that the use of mASM method achieves greater accuracy compared to ASM, when the input data set contains categorical variables.

## Data Availability

The datasets generated and/or analysed during the current study are available in the Gdrive repository, https://docs.google.com/spreadsheets/d/16t8wj1u8iGG57PIjgiVH7eGTY-t_XXe6/edit?usp=sharing&ouid=115675765202796328325&rtpof=true&sd=true.

## Abbreviations

AI: Artificial intelligence
QoE: Quality of experience
IF: Influence factor
ASM: Active subspaces method
mASM: Modified active subspaces method
STD: Standard deviation
HIF: Human IF
SIF: System IF
CIF: Context IF
QoS: Quality of service
PCA: Principal component analysis
MOS: Mean opinion score
SVD: Singular value decomposition
LSI: Latent semantic analysis
LPP: Locality preserving projections
ICA: Independent component analysis
PP: Projection pursuit
MDS: Multidimensional scaling
LLE: Locally linear embedding
SOM: Self-organizing map
t-SNE: T-distributed stochastic neighbor embedding
LDA: Linear discriminant analysis
LVQ: Learning vector quantization
SNE: Stochastic neighbor embedding
ASFS: Activity scores for feature selection
ASFE: Activity scores for feature extraction
VSFS: Variance/STD scores for feature selection
VSFE: Variance/STD scores for feature extraction
MAE: Mean absolute error
RMSE: Root mean square error
PCC: Pearson correlation coefficient
SROCC: Spearman rank order correlation coefficient
